# Effects of Garlic on Glucose Parameters and Lipid Profile: A Systematic Review and Meta-Analysis on Randomized Controlled Trials

**DOI:** 10.3390/nu16111692

**Published:** 2024-05-29

**Authors:** Xinyu Zhao, Tao Cheng, Hui Xia, Yanhong Yang, Shaokang Wang

**Affiliations:** 1 Key Laboratory of Environmental Medicine and Engineering of Ministry of Education, Department of Nutrition and Food Hygiene, School of Public Health, Southeast University, Nanjing 210009, China; 220223670@seu.edu.cn (X.Z.); huixia@seu.edu.cn (H.X.); 220223701@seu.edu.cn (Y.Y.); 2 Department of General Surgery, Zhongda Hospital of Southeast University, Nanjing 210009, China; chengtao860714@163.com; 3 Clinical Medical Research Center for Plateau Gastroenterological Disease of Xizang Autonomous Region, School of Medicine, Xizang Minzu University, Xianyang 712082, China

**Keywords:** garlic, glucose parameters, lipid profile, randomized controlled trials, meta-analysis

## Abstract

(1) Background: The effect of garlic on glucose and lipid metabolism in humans remains controversial. The aim of this study was to investigate the effects of garlic on blood lipid levels and glucose levels in humans through a systematic review and meta-analysis. (2) Methods: We extensively searched four databases, including PubMed, Web of Science, Embase, and the Cochrane Library, up to February 2024. To assess the collective impact of garlic and its supplements on fasting blood glucose (FBG), glycosylated hemoglobin (HbA1c), total cholesterol (TC), high-density lipoprotein cholesterol (HDL-C), low-density lipoprotein cholesterol (LDL-C), and triglycerides (TG), an analysis was conducted using a random effects model. Subgroup analyses were performed when *I*^2^ < 50%. (3) Result: We found that the garlic intervention was effective in controlling FBG (mean difference = −7.01; 95% CI: −8.53, −5.49, *p* < 0.001), HbA1c (mean deviation = −0.66; 95% CI: −0.76, −0.55, *p* < 0.001, *I*^2^ = 62.9%), TC (mean difference = −14.17; 95% CI: −19.31, −9.03, *p* < 0.001), and LDL-C (mean difference = −8.20; 95% CI: −15.58, −0.81, *p* = 0.03); moreover, it also increased the level of HDL-C in humans (mean difference = 2.06; 95% CI: 1.54, 2.59; *p* < 0.001). Nonetheless, the intervention involving garlic did not yield a substantial impact on triglyceride (TG) levels. (4) Conclusion: The intervention of garlic is beneficial to control blood glucose and blood lipids in humans.

## 1. Introduction

Many chronic non-communicable diseases, such as cardiovascular diseases, chronic respiratory diseases, cancers and diabetes, are the leading cause of death worldwide [1], causing 41 million deaths annually. Glucose and lipids are essential nutrients that provide energy to cells. In healthy individuals, glucose and lipid metabolism is precisely regulated [2]. Disorders of glucose and lipid metabolism can lead to a number of chronic diseases, including atherosclerosis, diabetes and fatty liver disease [3]. It is an important pathological feature of many chronic diseases. Dyslipidemia, including elevated TC, LDL-C and TG and reduced HDL-C, is an important cause of cardiovascular disease [4], particularly coronary events and atherosclerosis. Chronic liver disease may also increase the risk of cardiovascular events [5]. According to the World Health Organization (WHO), the most important risk factors for cardiovascular disease are hypertension, high cholesterol, alcohol consumption, smoking and obesity [6]. Elevated FBG and HbA1c are characteristic of type 2 diabetes mellitus (T2DM) [7]. Therefore, glycolipid metabolic parameters such as blood glucose, cholesterol and triglycerides are important indicators of an individual’s metabolic health. At present, most of the clinical treatments for glycolipid metabolic diseases are symptomatic and focused on symptomatic relief, such as insulin therapy, and the drug treatments are prone to toxic side effects, which bring pain to the patients. Therefore, it has become a research hotspot to find effective substances from natural products with little toxic side effects to treat glycolipid metabolic diseases.

Garlic, commonly used in traditional medicine as a flavoring agent and functional food as well as a traditional medicine, has a wide range of biological effects, with a host of functions such as anticancer, antioxidant, antimicrobial, anti-mutagenic, anti-asthmatic, immunomodulatory, and prebiotic functions [8,9]. These characteristics in garlic arise from the presence of sulfur and a variety of compounds, such as allicin, alliin and methyl allyl trisulfide (MATS) [10,11]. A study proves that garlic is an effective herbal plant for controlling blood sugar [12], Similarly, the hypoglycemic effect of garlic has been demonstrated in animal models [13]. The hypolipidemic effect of garlic involves a complex mechanism, and studies have found that a variety of garlic extracts play a role in lowering blood lipids through different mechanisms: Lin XL et al. found that allicin-induced up-regulation of ATP Binding Cassette Subfamily A Member 1 (ABCA1) could enhance cholesterol efflux and decrease lipid accumulation by activating the PPARγ/LXR α signaling pathway [14]. Another study showed that garlic powder extract (GPE) inhibits ACAT activity and enhances cholesteryl ester hydrolase (CEH) activity, which significantly reduces the accumulation of intracellular cholesteryl esters [15]. In a study by Hwang YP et al., S-allyl cysteine (SAC) activated adenylate-activated protein kinase AMP-activated protein kinase (AMPK) through calcium/calmodulin-dependent kinase calcium/calmodulin-dependent kinase (CaMKK), silencing of the information regulator T1, and inhibition of hepatic lipogenesis mediated by sterol regulatory element-binding protein-1 (SREBP-1) [16]. 

Therefore, the present study aimed to investigate the effects of garlic on blood glucose levels and lipid parameters in humans through systematic evaluation and meta-analysis. It provides new ideas for the development of natural products against diseases related to glycolipid metabolism.

## 2. Materials and Methods

The study was designed in accordance with the recommended guidelines of Reporting Items for Systematic Reviews and Meta-Analysis (PRISMA) 2020.

### 2.1. Literature Search Strategy 

To examine the impacts of garlic on lipid profiles and glycemic parameters, a thorough review of the literature was performed. Four electronic databases including PubMed, Embase, Cochrane Library and Web of Science were searched from the time of database creation to February 2024. Search terms included the following: (garlic OR Allium sativum OR allium) AND (Blood Glucose OR Blood Sugar OR Sugar, Blood OR Glucose, Blood OR glucose blood level OR Dyslipidemias OR Dyslipidemia OR Dyslipoproteinemias OR Dyslipoproteinemia OR Lipids OR lipid OR cholesterol) AND (randomized controlled trial OR clinical trial, randomized OR controlled clinical trial OR randomized, and trial OR randomized OR intervention OR random OR controlled trial OR placebo). In addition, reference lists of relevant review articles and retrieved studies were hand-searched for eligible trials that might have been missed. Endnote X9 was used for document management.

### 2.2. Inclusion and Exclusion Criteria

The inclusion criteria for this study were as follows: (1) randomized clinical trials with a parallel or crossover design and an experimental period exceeding 2 weeks; and (2) inclusion of at least one of the following outcome variables: HbA1c, FBG, TC, HDL-C, LDL-C, and TG; (3) the study subjects were adults aged ≥ 18 years; (4) the control group received a placebo.

Any clinical trial meeting any of the following criteria was excluded: (1) use of interventions other than garlic; (2) combination of garlic interventions with other food supplements; (3) study participants that were pregnant women; (4) cytological studies, animal experiments, or non-clinical studies; and (5) trials with incomplete or irrelevant data.

### 2.3. Data Extraction 

Publications and data were independently screened and extracted by two authors, extracting the following data: the first author’s name, year of publication, sample size, participant, country, age range, follow-up period and type of garlic as an intervention. In addition, from each literature source, the mean and standard deviation of blood glucose control indicators (HbA1c and FBG) and blood lipid control indicators (TC, LDL-C, HDL-C, and TG) were extracted. In cases where these values were not provided, conversions were made using available data such as 95% Cis, standard error of the mean (SEM), or median values.

### 2.4. Quality Assessment 

The quality of included studies was assessed using tools from the Cochrane Collaboration (Cochrane Collaboration) [17]. Each study was assessed for randomized sequence generation, allocation concealment, subject blinding, personnel and outcome assessment, incomplete outcome data, and selective reporting. Studies were categorized as low risk of bias, high risk of bias or unclear in each area.

### 2.5. Data Analysis

To convert IFCC units for glycated hemoglobin (mmol/mol) to NGSP units (%), we used the formula NGSP = 0.0915 × IFCC + 2.15%. We also converted blood glucose and lipid levels from various units to mg/dL, with 1 mmol/L TC, LDL-C, and HDL-C equaling 38.7 mg/dL, 1 mmol/L TG equaling 88.5 mg/dL, and 1 mmol/L FBG equaling 18 mg/dL. Our data were obtained from literature sources and analyzed using Stata SE 15.1 software.

Mean changes in lipid parameters, blood glucose levels, and glycated hemoglobin levels were determined using continuous variables. These changes were calculated based on the baseline and endpoint data. The formula used to calculate the mean difference (MD) and standard deviation (SD) of each outcome variable before and after intervention was as follows: SD _change_ = (SD^2^ _baseline_ + SD^2^ _endpoint−2_ × R × SD _baseline_ × SD _endpoint_) 1/2, where R is the correlation coefficient set at 0.5 [18]. To evaluate the heterogeneity of the studies, a chi-square test and *I*^2^ index were conducted. If the *p*-value of the chi-square test was less than 0.10 or *I*^2^ was greater than 50%, significant heterogeneity among the studies was considered to exist, and a random-effects model was used. The threshold for statistical significance was set at 0.05. Subgroup analyses based on garlic type, participant type, and duration were conducted to explore possible sources of heterogeneity.

In our study, we employed sensitivity analysis to evaluate the impact of individual studies on the overall findings. To detect potential publication bias, we utilized funnel plots and conducted Egger’s test. For indicators with more than 10 publications, we quantitatively assessed publication bias using funnel plots and Egger’s test. Additionally, we employed trim and fill analysis (metatrim) to evaluate the influence of publication bias on the stability of the results. 

## 3. Result

### 3.1. Process of Study Selection

**Figure 1:**
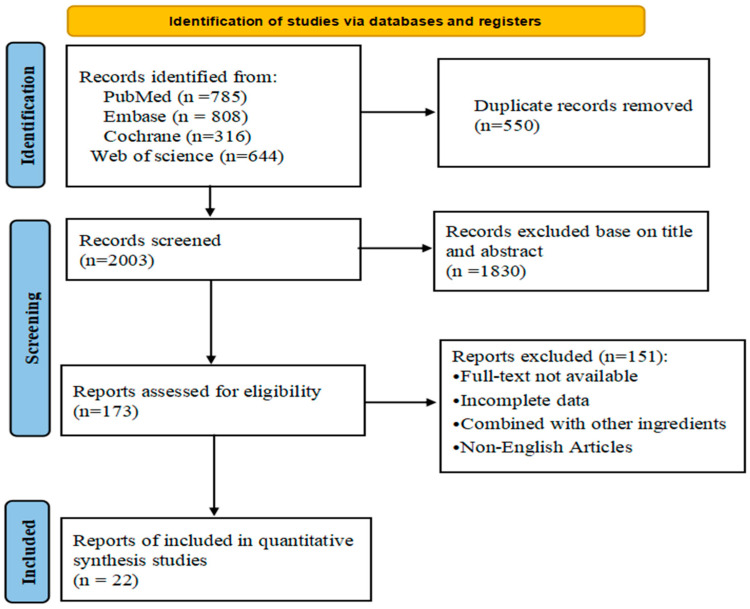
PRISMA flow chart of selected articles.

A comprehensive search of four databases yielded a total of 2553 relevant papers. Among these, 550 duplicate publications were identified and excluded. Through a careful evaluation of titles and abstracts, 1830 papers that were deemed irrelevant to our study were also excluded. The remaining 173 papers underwent a thorough qualification review. From this review, 151 papers were excluded due to various reasons, including incomplete data, unavailability of full text, the use of cinnamon in combination with other drugs, or the absence of a placebo in the control group. Ultimately, our analysis included 22 pieces of literature, comprising 29 trials. The flowchart illustrating the literature search process is presented in Figure 1.

### 3.2. Study Characteristics

Table 1 presents the fundamental attributes of the studies integrated into this meta-analysis. A total of 1567 participants from Canada [19], Iran [20,21,22,23], Pakistan [24,25,26], India [27,28,29], the United States [30,31,32,33], Korea [34], Israel [35], Russia [36,37], Poland [38], Brazilian [39] and Denmark [40] were included. The average age of the subjects in each trial varied, ranging from 18 to 80 years. The intervention period for garlic ranged from 3 weeks to 1 year. The 22 articles included participants with various health conditions, such as hyperlipidemia, hemodialysis, type 2 diabetes, myocardial infarction, coronary artery disease, non-alcoholic fatty liver disease, obesity, hypertension, polycystic ovary syndrome, and healthy adults. Most of the subjects did not receive any medication during the study period, while some continued their daily medication throughout the trial. Garlic powder was used in 17 trials, while garlic oil, aged garlic extract, raw garlic, and enteric garlic supplements were used in 7 trials. The daily doses varied depending on the type of preparation, with garlic powder ranging from 300 to 22,400 mg/day, garlic oil at 4000 mg/day, aged garlic extract at 1200–6000 mg/day, raw garlic at 4000 mg/day, and enteric-coated garlic supplements at 800 mg. The dosages of the garlic preparations were not directly comparable due to differences in active ingredient and bioavailability between the different types of preparations (powder, oil, aged extract, and raw). Additionally, the dosage of the active ingredient may also vary within a type, depending on the brand and processing method.

### 3.3. Risk of Bias Assessment

Table 2 displays the assessment of bias risk. The majority of the studies were deemed to have a minimal risk of bias.

### 3.4. Results of Meta-Analysis

#### 3.4.1. Effect of Garlic on Indicators Related to Glucose Metabolism

##### Impact of Garlic on Glucose Parameters

**Figure 2:**
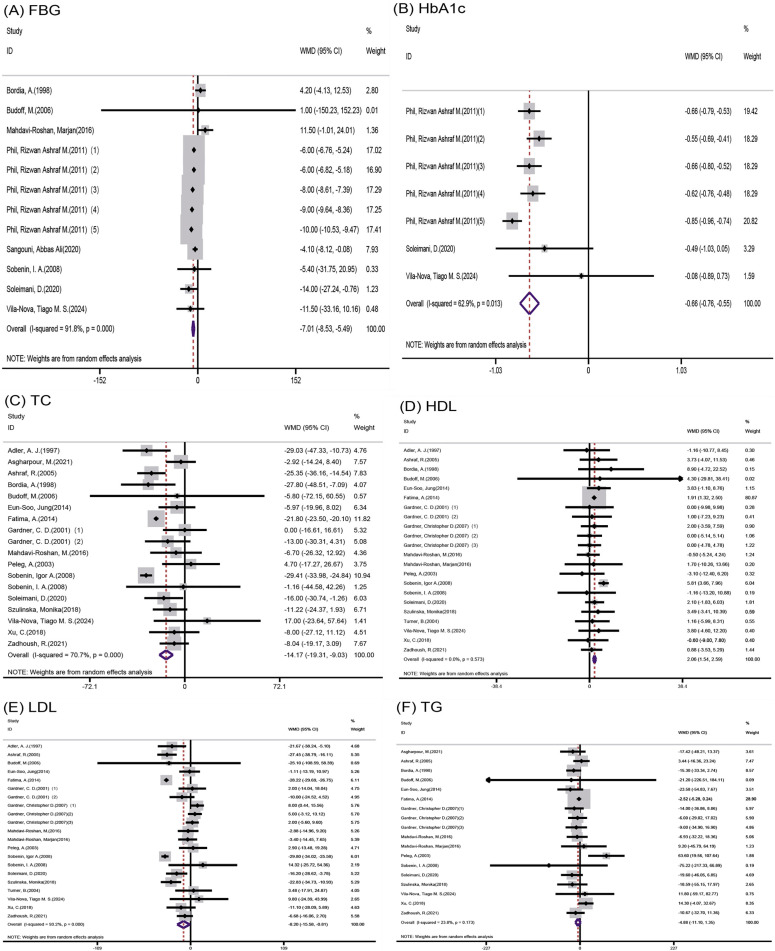
Forest plot of effects of (**A**) FBG = fasting blood glucose, (**B**) HbA1c = glycosylated hemoglobin, (**C**) TC = total cholesterol, (**D**) HDL-C = high-density lipoprotein cholesterol, (**E**) LDL-C = low-density lipoprotein cholesterol, and (**F**) TG = triglyceride levels [19,20,21,22,23,24,25,26,27,28,29,30,31,32,33,34,35,36,37,38,39,40].

Among the studies focusing on FBG, 8 eligible studies with 12 effect sizes were examined. The garlic intervention demonstrated a significant influence on FBG levels (mean deviation = −7.01; 95% CI: −8.53, −5.49, *p* < 0.001, *I*^2^ = 91.8%, as shown in Figure 2A).

Likewise, HbA1c levels were assessed across three trials with seven effect sizes; outcomes from the random-effects model revealed a noteworthy impact of the garlic intervention on HbA1c levels (mean deviation = −0.66; 95% CI: −0.76, −0.55, *p* < 0.001, *I*^2^ = 62.9%, as illustrated in Figure 2B).

#### 3.4.2. Impact of Garlic o Lipid Profile

The analysis encompassed 19 effect sizes from 17 studies to evaluate serum total cholesterol levels, revealing a noteworthy decrease attributed to garlic intervention (mean difference = −14.17; 95% CI: −19.31, −9.03, *p* < 0.001, *I*^2^ = 70.7%, Figure 2C).

Exploration into the impact of garlic supplementation on HDL-C levels involved 19 studies with 22 effect sizes, demonstrating a significant positive influence (mean difference = 2.06; 95% CI: 1.54, 2.59, *p* < 0.001, *I*^2^ = 0.0%, Figure 2D).

Pooling data from 18 randomized controlled trials comprising 21 effect sizes, the meta-analysis indicated a substantial reduction in LDL-C concentrations due to garlic intake (mean difference = −8.20; 95% CI: −15.58, −0.81, *p* = 0.03, *I*^2^ = 93.2%, Figure 2E).

Evaluation of 18 effect sizes from 16 studies did not reveal a statistically significant impact of garlic intervention on TG levels (mean difference = −4.88; 95% CI: −11.1, 1.35, *p* = 0.125, *I*^2^ = 23.8%, Figure 2F).

### 3.5. Subgroup Analysis

In cases where heterogeneity surpasses the 50% threshold, the reliability of the combined outcomes may be compromised, necessitating a critical evaluation of the appropriateness of these results. Subgroup analysis becomes imperative to identify the origins of such heterogeneity. Notably, for FBG, HbA1C, TC, and LDL, all exhibiting heterogeneity levels exceeding 50%, we proceeded with subgroup analysis. Regrettably, the limited availability of literature pertaining to HbA1C precluded its inclusion in the analysis. Heterogeneity disappeared for FBG (*I*^2^ = 0.0%, *p* = 0.873) when grouped by type of garlic intervention (AGE and Other), and TC (*I*^2^ = 0.0%, *p* = 0.781) when grouped by type of garlic, and similarly for LDL (*I*^2^ = 49.4%, *p* = 0.095) when grouped by type of intervention. We also grouped analyses by population type (hyperlipidemia, type 2 diabetes mellitus, healthy, other) and heterogeneity was reduced for FBG (*I*^2^ = 36.6%, *p* = 0.177), TC (*I*^2^ = 0.0%, *p* = 0.546) and LDL (*I*^2^ = 29.8%, *p* = 0.200). We also analyzed the duration subgroups with reduced heterogeneity in FBG (*I*^2^ = 22.5%, *p* = 0.256), as evidenced by reduced TC heterogeneity (*I*^2^ = 0.0%, *p* = 0.919) and similarly reduced LDL heterogeneity (*I*^2^ = 0.0%, *p* = 0.669). 

The FBG lowering treatment effect was more pronounced in trials with longer durations (subgroup > 8 weeks: mean difference  =  −7.29, 95% CI: −8.78, −5.80, *I*^2^ =  92.7%; *p* <  0.001), among Other garlic intervention types (subgroup =Other: mean difference  =  −6.98, 95% CI: −8.51, −5.44, *I*^2^ =  93.3%; *p* <  0.001), in T2DM (subgroup =T2DM: mean difference  =  −7.01, 95% CI: −8.53, −5.49, *I*^2^ =  95.5%; *p* <  0.001).

The therapeutic effect of lowering TC was more pronounced in trials with longer durations (subgroup >8 weeks: mean difference = −16.86, 95% CI: −22.20, −11.52, *I*^2^= 66.8%; *p* < 0.001); among Other garlic intervention types (subgroup = Other: mean difference  =  −14.93, 95% CI: −20.40, −9.45, *I*^2^ =  72.5%; *p* <  0.001), in T2DM (subgroup =T2DM: mean difference  =  −28.54, 95% CI: −32.73, −24.34, *I*^2^ =  0.0%; *p* <  0.368).

The therapeutic effect of reducing LDL was more significant in trials with longer durations (subgroup >8 weeks: mean difference = −9.04, 95% CI: −17.25, −0.83, *I*^2^ = 93.9%; *p* < 0.001), in T2DM (subgroup = T2DM: mean difference  =  −26.11, 95% CI: −36.81, −15.42, *I*^2^ =  57.5%; *p* <  0.095). The results are shown in Table 3.

### 3.6. Publication Bias and Sensitivity Analysis

**Figure 3:**
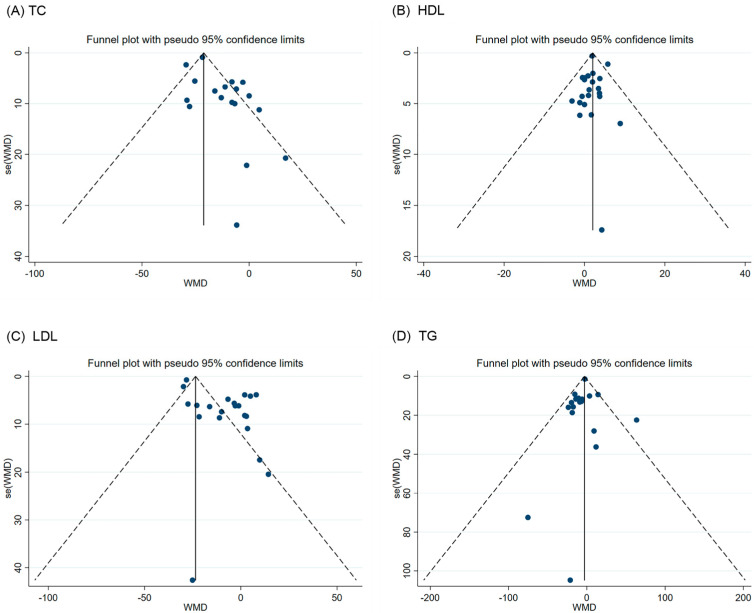
Funnel plot and Egger test for assessing publication bias. (**A**) TC Egger’s test: *p* = 0.011; (**B**) HDL-C Egger’s test: *p* = 0.765; (**C**) LDL-C Egger’s test: *p* = 0.001; (**D**) TG Egger’s test: *p* = 0.504. TC (total cholesterol), HDL-C (high-density lipoprotein cholesterol), LDL-C (low-density lipoprotein cholesterol), TG (triglyceride).

Funnel plots and Egger’s test were used to assess whether studies had publication bias. The points in the funnel plot indicate the included studies. As shown in Figure 3, the results of the funnel plot and Egger’s test indicated that there was no publication bias in the studies except for TC and LDL (FBG Egger’s test: *p* = 0.285; HbA1C Egger’s test: *p* = 0.209; HDL Egger’s test: *p* = 0.765; TG Egger’s test: *p* = 0.504). 

**Figure 4:**
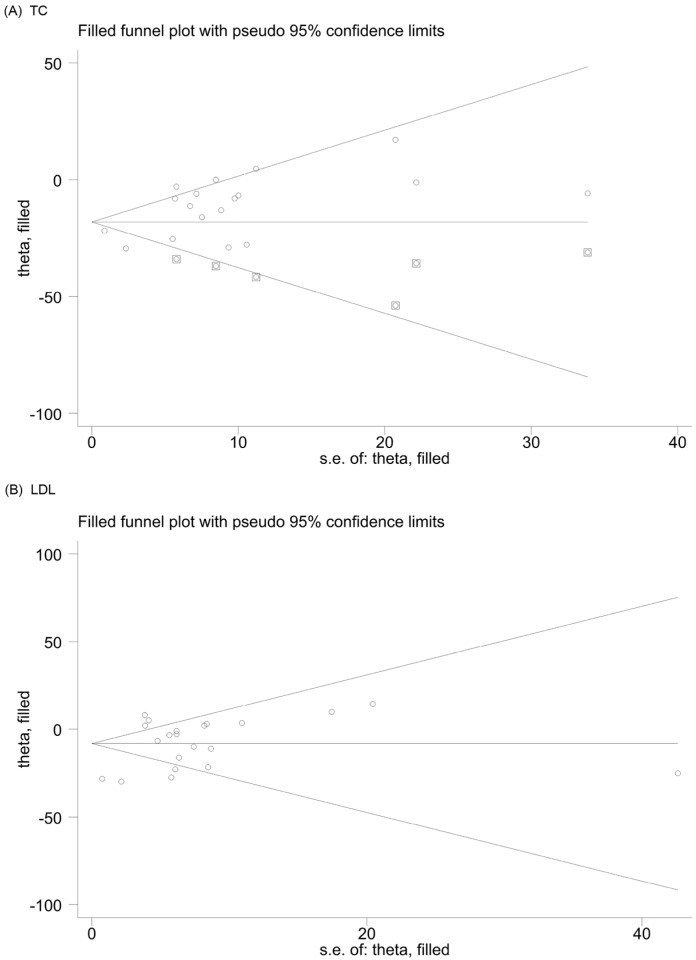
The funnel plot filled with effect estimates by standard error in means was constructed for TC (**A**) and LDL (**B**). TC = total cholesterol; LDL-C = low-density lipoprotein cholesterol.

Therefore, we further assessed the stability of TC results using the trim and fill analysis (metatrim). After populating the six dummy documents, the *p*-values before and after trimming and populating the analyses were less than 0.05. Similarly, the trim and fill analysis for LDL levels showed that no padding was performed, and the data were unchanged. The *p* values before and after the trim and fill analysis were less than 0.05. All of the above results indicate that the results were robust. Thus, although there was publication bias in the TC and LDL data, it had little effect on the stability of the results (Figure 4).

**Figure 5:**
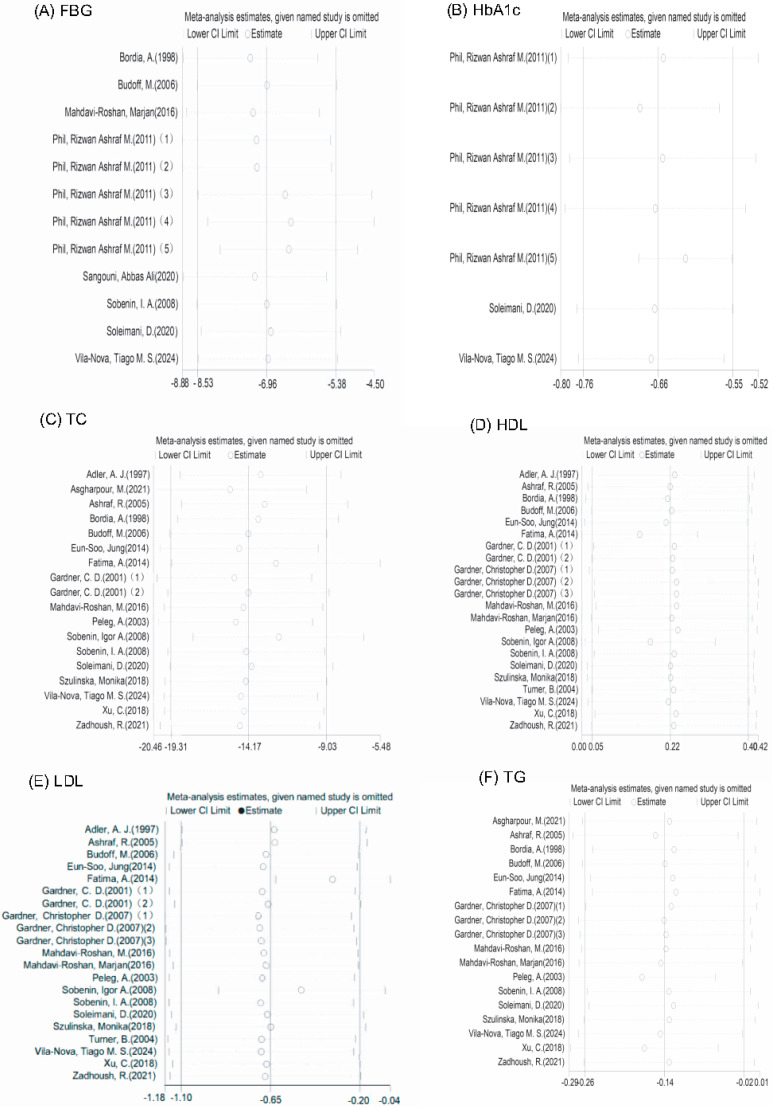
Sensitivity analysis. (**A**) FBG, (**B**) HbA1c, (**C**) TC, (**D**) HDL-C, (**E**) LDL-C, and (**F**) TG. Definitions: FBG (fasting blood glucose), HbA1c (glycosylated hemoglobin), TC (total cholesterol), HDL-C (high-density lipoprotein cholesterol), LDL-C (low-density lipoprotein cholesterol), TG (triglyceride) [19,20,21,22,23,24,25,26,27,28,29,30,31,32,33,34,35,36,37,38,39,40].

We conducted sensitivity analyses to assess the influence of each study on the overall effect size by systematically excluding one study at a time from the analysis. Our findings revealed that no single trial significantly affected the overall effect sizes of FBG, HbA1c, TC, LDL-C, HDL-C, and TG (Figure 5).

## 4. Discussion

The aim of this systematic evaluation was to investigate the effect of garlic on glycolipid metabolism in adults, and 22 RCTs were included; we found that garlic significantly modulated FBG, HbA1c, TC, LDL, and HDL in patients but had no effect on TG levels. Due to the high heterogeneity of findings, a random effects model was used in all of the following stages. The duration of the intervention period for the studies included in the meta-analysis was 3 weeks−1 year, and the types of garlic included raw garlic, aged garlic extract, and garlic powder tablets. The results showed that garlic has a beneficial effect on blood glucose and blood lipid in humans, and their association was statistically significant.

The functional food industry has seen considerable growth over the years; as a result, products developed from garlic have emerged. Garlic products widely used in the market mainly include garlic oil, aged garlic extract, black garlic, garlic powder, etc. [41]. All of these products were also used in the literature included in the meta-analysis. The main active components of garlic are its organosulfur compounds, including E/Z-ajoene, S-allyl cysteine, S-allyl cysteine sulfoxide (alliin), diallyl thiosulfinate (allicin), diallyl sulfide, diallyl disulfide, diallyl trisulfide, and allyl methyl trisulfide [42,43]. Previous meta-analyses have shown that garlic interventions have a beneficial effect on lowering blood glucose levels, and our findings are consistent with this [44,45,46]. According to Thomson’s findings, the use of high doses of garlic extract (500 mg/kg) significantly reduced blood glucose levels in normal rats in a study that lasted 4 weeks [47]. In 2005, Demerdash studied the hypoglycemic effect of using garlic extract in the treatment of tetracycline-induced diabetic rats. Serum glucose levels in rats were reduced by daily intake of garlic extract at a dose of 4 g/kg for 4 weeks [48]. It has been suggested in the literature that the antihyperglycemic effect of garlic may be associated with increased insulin secretion [49]. Previous studies have indicated that in diabetic rats, garlic functions as a stimulant for insulin secretion, and the anti-diabetic attributes of SAC sulfoxide may be further enhanced by its antioxidative properties [50]. Collectively, garlic demonstrates the capacity to mitigate complications associated with diabetes [49]. Homeostasis model assessment (HOMA) also evaluates insulin sensitivity, insulin resistance, and more. An animal study showed that garlic supplementation reduced the homeostasis model assessment of insulin resistance (HOMA-IR) in high-fat-fed mice [51]. A randomized controlled clinical trial showed that supplementation with garlic powder for 3 months improved the insulin and HOMA-IR status in NAFLD patients [21]. Previous meta-analyses have also shown a significant effect of garlic on HOMA-IR reduction [52].

Long-term glycemic control is accurately represented by HbA1c. Research indicates that even a 1% decrease in HbA1c significantly lowers the likelihood of diabetes-related issues, including peripheral vascular disease, microvascular complications, myocardial infarction, and stroke [53]. The WHO advises the following: “HbA1c can be used as a diagnostic test for diabetes, provided that rigorous quality assurance testing is performed, the assay is standardized according to criteria consistent with international references, and there are no conditions that prevent its accurate measurement. These conditions include pregnancy, suspected type 1 diabetes, short duration of diabetes symptoms, acute illness, treatment with medications that may cause rapid increases in blood glucose levels, pancreatic injury, haemoglobinopathies, anemia, renal failure and HIV infection” [54]. The significance of managing HbA1c levels in individuals with diabetes is underscored by this demonstration. Our research demonstrated a notable impact of garlic on HbA1c levels, aligning with the conclusions drawn from prior meta-analyses [45].

Our study showed that garlic consumption had a significant effect on TC, LDL and HDL. Garlic’s ability to modulate TC, LDL, HDL and TG has been linked to various conditions stemming from disrupted lipid metabolism. These include nonalcoholic fatty liver disease, coronary artery disease, and the development of atherosclerosis [55,56]. This might have been possible due to the presence of the allicin compound and its derivatives by inhibiting the HMG–CoA reductase enzyme [35,57,58]. In addition, the active ingredient SAC in garlic attenuated free-fatty-acid-induced adipogenesis in human HepG2 cells by activating an AMP-activated protein kinase dependent pathway [16]. Studies have shown that aged garlic extract can improve insulin resistance, which may be related to its effect on the gut microbiota [59]. Maha and his team’s study noted that adding 8 percent fresh garlic to the diet of rats was effective in reducing TC and LDL-C levels [60]. Research in Warshafsk suggests that garlic intake can reduce cholesterol levels by about 10% [61]. In another study conducted by Rahmani, TC levels and LDL-C were reduced by supplementing garlic powder for 12 weeks [62]. Similarly, supplementation of a 400 mg/day dose of garlic in patients with cardiovascular diseases resulted in a decrease in TC, TG and LDL levels and a significant increase in HDL-C levels [63]. Our study did not find a significant effect of garlic on TG. It may be the effect of different types of garlic or other factors such as intervention dose.

Acknowledging certain limitations of this study is crucial. Initially, the intervention approaches involving garlic differed across the 22 articles encompassed in our analysis. Its active ingredients also varied. The main components of garlic include allyl alkynes and allyl enzymes. Garlic powder contains three active substances, allyl, alkaline and allicin. Old garlic is rich in antioxidant compounds, including allicin and selenium [64,65]. The results of subgroup analyses showed that FBG and TC had significant effects in all types except AGE. It is important to note that the reasons for heterogeneity can only be partially explained by the variables explored in the subgroup analyses. The fact that there was still a group with high heterogeneity after the subgroup analyses implies that there are other factors that could explain the high heterogeneity between studies. Second, intervention dose and duration varied across the included studies, but because of the different types of interventions, we did not conduct more detailed analyses to determine potential effects. In addition, because the subjects in each trial had different types of disease, their lifestyles may have been different and, therefore, would have had different effects. Finally, the included studies were highly heterogeneity. It is worth noting that there was some publication bias in this study, possibly due to the researcher’s tendency to publish positive results. Therefore, more randomized controlled trials with larger numbers of participants and better designs are needed.

## 5. Conclusions

In conclusion, this meta-analysis suggests that garlic intervention improves some lipid indices in patients and outperforms the control group in various aspects, and it can be surmised that garlic therapy should be beneficial for patients with disorders related to glucose and lipid metabolism. However, there was no significant difference in the effect of garlic intervention on patients’ TG levels, and larger sample size trials in other populations may be needed to confirm these findings.

## Figures and Tables

**Table 1:** Baseline characteristics of the studies included.

Study	Year	Study Region	Sample Size	Comorbidities	Dose of Product (mg/Day)	Dose of Active Ingredient per Day	Treatment Duration	Age Range	Type of Intervention(I/C)	Drug Use during the Study Period
Adam J Adler [19]	1997	Canada	23	hyperlipidemia	900	NA	12 weeks	NA	garlic pill/placebo	other drugs that do not affect blood lipids
Asgharpour, M. [20]	2021	Iran	140	hemodialysis	600	2.6 mg garlic extract	8 weeks	18–70	garlic powder/placebo	conventional drugs
Ashraf, R. [24]	2015	Pakistan	70	T2DM	600	7.8 mg alliin	12 weeks	25–70	garlic powder tablets/placebo	NA
A. Bordia [27]	1998	India	60	fold healed myocardial infarction	4000	NA	3 months	NA	garlic oil preparations/placebo	nitrates and aspirin
Budoff Matthew [30]	2006	US	19	CAD	4 mL/d	NA	1 year	NA	AGE/placebo	statin (10–40 mg/day)
Eun-Soo, Jung [34]	2014	Korea	55	hyperlipidemia	6000/d	3 mg/g SAC	12 weeks	NA	AGE/placebo	NA
AJAZ FATIMA [25]	2014	Pakistan	106	hyperlipidemia	900 mg	NA	3 months	20–70	garlic tablets/placebo	NA
Gardner, C. D. [31]	2001	US	51	hyperlipidemia	1000/500	1.5 mg allicin/0.75 mgallicin	12 weeks	35–65	garlic tablets/placebo	NA
Gardner, Christopher D. [33]	2007	US	192	hyperlipidemia	4000/4000/4000	NA/3.2 mg allicin/1.5 mg SAC	6 months	NA	raw garlic/garlic tablets/placebo	NA
Mahdavi-Roshan, M. [28]	2016	Iran	56	CAD	800	2.4 mg allicin	3 months	56	garlic powder tablets/placebo	prescribed medications
Mahdavi-Roshan, Marjan [29]	2016	Iran	24	Healthy	2400	2400 mg allicin	3 weeks	25–55	garlic powder tablets/placebo	NA
Peleg, A. [35]	2003	Israeli	33	hyperlipidemia	22400	22,400 mg alliin	16 weeks	18–80	garlic powder/placebo	NA
Rizwan Ashraf M. Phil [26]	2011	Pakistan	180	T2DM	300/600/900/1200/1500	NR	24 weeks	NA	garlic tablets/placebo	NA
Abbas Ali Sangouni [21]	2020	Iran	88	NAFLD	1600	6 mg allicin	3 months	>18	garlic powder tablets/placebo	NA
Igor A. Sobenin [36]	2008	Russia	42	T2DM	600	NA	12 weeks	35–70	garlic powder tablets/placebo	NA
Igor A. Sobenin [37]	2008	Russia	20	T2DM	600	NA	28 days	34–62	garlic powder Tablets/placebo	NA
Soleimani, D. [22]	2020	Iran	98	NAFLD	800	1.5 mg allicin	15 weeks	20–70	enteric garlic powder/placebo	conventional treatment medications
Szulinska, Monika [38]	2018	Poland	92	Obesity	400	8 mg alliin	3 months	25–60	garlic extract capsules/placebo	NA
Turner, B. [40]	2004	Denmark	62	Healthy	920	9 mg alliin	12 weeks	40–60	garlic powder tablets/placebo	NA
Vila-Nova, Tiago M. S. [39]	2024	Brazil	28	hypertensive	1200	1.2 mg SAC	12 weeks	19–59	AGE/placebo	NA
Xu, C. [32]	2018	US	48	Obesity	3600	NA	6 weeks	25–65	AGE/placebo	NA
Zadhoush, R. [23]	2021	Iran	80	PCOS	800	NA	8 weeks	18–45	garlic pills/placebo	NA

Abbreviation: T2DM = diabetes mellitus type 2; CAD = coronary artery disease; NAFLD = non-alcoholic fatty liver disease; PCOS = polycystic ovary syndrome; AGE= aged garlic extract; SAC= S-allylcysteine; NA = not available.

**Table 2:** Risk of bias assessment.

Study	Random Sequence Generation	Allocation Concealment	Blinding of Participants and Personnel	Blinding of Outcome Assessment	Incomplete Outcome Data	Selective Reporting	Other Bias
Adam J Adler [19]	unclear	unclear	low	unclear	low	low	unclear
Asgharpour, M. [20]	low	low	low	unclear	low	low	unclear
Ashraf, R. [24]	unclear	unclear	unclear	unclear	low	low	unclear
A. Bordia [27]	unclear	unclear	low	unclear	low	low	unclear
Budoff Matthew [30]	unclear	unclear	low	low	low	low	unclear
Eun-Soo, Jung [34]	unclear	unclear	low	unclear	low	low	unclear
AJAZ FATIMA [25]	unclear	unclear	low	unclear	low	low	unclear
Gardner, C. D. [31]	unclear	low	low	unclear	low	low	unclear
Gardner, Christopher D. [33]	unclear	low	low	unclear	low	low	unclear
Mahdavi-Roshan, M. [28]	unclear	low	unclear	unclear	low	low	unclear
Mahdavi-Roshan, Marjan [29]	unclear	unclear	low	unclear	low	low	unclear
Peleg, A. [35]	unclear	unclear	low	unclear	low	low	unclear
Rizwan Ashraf M. Phil [26]	unclear	unclear	low	unclear	low	low	unclear
Abbas Ali Sangouni [21]	low	unclear	low	unclear	low	low	unclear
Igor A. Sobenin [36]	unclear	unclear	low	unclear	low	low	unclear
Igor A. Sobenin [37]	unclear	unclear	low	unclear	low	low	unclear
Soleimani, D. [22]	unclear	unclear	low	low	low	low	unclear
Szulinska, Monika [38]	unclear	unclear	low	unclear	low	low	unclear
Turner, B. [40]	unclear	unclear	low	unclear	low	low	unclear
Vila-Nova, Tiago M. S. [39]	low	unclear	unclear	unclear	low	low	unclear
Xu, C. [32]	unclear	unclear	low	unclear	low	low	unclear
Zadhoush, R. [23]	low	low	low	unclear	low	low	unclear

**Table 3:** The results of subgroup analysis.

Index	Subgroup	Mean Difference		*p*	*I*^2^ (%)	*p* Value of Heterogeneity
		Mean	95% CI			
	Type of intervention					
FBG	AGE	−11.25	−32.69, 10.19	0.304	0.0	0.873
	Other *	−6.98	−8.51, −5.44	0.000	93.3	0.000
TC	AGE	−7.49	−15.81, 0.83	0.078	0.0	0.781
	Other	−14.93	−20.40, −9.45	0.000	72.5	0.000
LDL	AGE	−9.91	−22.00, 2.19	0.108	49.4	0.095
	Other	−7.95	−16.29, 0.38	0.061	94.9	0.000
	Population condition					
FBG	Hyperlipidemia	/	/	/	/	/
	T2DM	−7.01	−8.53, −5.49	0.000	95.5	0.000
	Healthy	/	/	/	/	/
	Other **	−3.83	−10.04, 2.38	0.227	36.6	0.177
TC	Hyperlipidemia	−12.23	−22.43, −2.04	0.019	72.5	0.003
	T2DM	−28.54	−32.73, −24.34	0.000	0.0	0.368
	Healthy	/	/	/	/	/
	Other	−9.24	−14.61, −3.88	0.001	0.0	0.546
LDL	Hyperlipidemia	−4.58	−18.79, 9.63	0.527	96.6	0.000
	T2DM	−26.11	−36.81, −15.42	0.000	57.5	0.095
	Healthy	−1.95	−11.77, 7.87	0.697	0.0	0.575
	Other	−10.97	−17.73, −4.21	0.001	29.8	0.200
	Duration					
FBG	≤8 week	7.19	−7.25, 21.63	0.329	22.5	0.256
	>8 week	−7.29	−8.78, −5.80	0.000	92.7	0.000
TC	≤8 week	−5.76	−12.99, 1.47	0.119	0.0	0.919
	>8 week	−16.86	−22.20, −11.52	0.000	66.8	0.000
LDL	≤8 week	−5.64	−12.14, 0.86	0.089	0.0	0.669
	>8 week	−9.04	−17.25, −0.83	0.031	93.9	0.000

* garlic pill, garlic powder, garlic oil preparations, garlic tablets, raw garlic, enteric garlic powder, garlic extract capsule. ** hemodialysis, fold healed myocardial infarction, CAD, NAFLD, Obesity, hypertensive, PCOS. Abbreviation: AGE = aged garlic extract; T2DM = diabetes mellitus type 2; CAD = coronary artery disease; NAFLD = non-alcoholic fatty liver disease; PCOS = polycystic ovary syndrome.
